# WDR7 up-regulation upon knocking down of neighboring non-coding RNA using siRNAs encapsulated in polyamidoamine dendrimers

**DOI:** 10.22038/ijbms.2019.36135.8607

**Published:** 2019-11

**Authors:** Sara Kor, Vahid Erfani-Moghadam, Reza Sahebi, Shabbou Bahramian, Mohammad Shafiee

**Affiliations:** 1 Stem Cell Research Center, Golestan University of Medical Sciences, Gorgan, Iran; 2 Medical Cellular and Molecular Research Center, Golestan University of Medical Sciences, Gorgan, Iran; 3 Department of Medical Nanotechnology, School of Advanced Technologies in Medicine, Golestan University of Medical Sciences, Gorgan, Iran; 4 Department of Modern Sciences and Technologies, Faculty of Medicine, Mashhad University of Medical Sciences, Mashhad, Iran; 5 Department of Molecular Medicine, School of Advanced Technologies, Shahrekord University of Medical Sciences, Shahrekord, Iran; 6 Department of Medical Genetics, School of Advanced Technologies in Medicine, Golestan University of Medical Sciences, Gorgan, Iran

**Keywords:** Breast cancer, lncRNA ROR, Polyamidoamine dendrimer, (PAMAM), Transfection, WDR7

## Abstract

**Objective(s):**

Breast cancer is the second leading cause of cancer death in females. Understanding molecular mechanisms in cancer cells compared with normal cells is crucial for diagnostic and therapeutic strategies. Long intergenic non-protein coding RNA, a regulator of reprogramming (lincRNA-RoR) is a noncoding RNA which initially was detected in induced pluripotent stem cells, and it has an important role in cell reprogramming and highly expressed in breast cancer cells. A key point in successful gene silencing is the usage of siRNA delivery system that is safe and efficient.

**Materials and Methods:**

In this study, the fifth-generation of PAMAM dendrimer is used as a nanocarrier for entering siRNA molecules for gene silencing of lincRNA-RoR. WDR7 is the gene encoding adjacent of lincRNA-RoR, which has an important role in apoptosis and cell cycle. Gel retardation assay was used to find the best Negative/Positive (N/P) molar charge ratio of siRNA- PAMAM transfected into MDA-MB 231 cells. MTT assay was performed 24 hr after transfection revealed the IC50 value (half maximal inhibitory concentrations) about 100 nanomolar for lincRNA-ROR siRNA.

**Results:**

The lincRNA-RoR and WDR7 gene expression changes were evaluated by real-time PCR after siRNA treatment and showed an increase in the gene expression of WDR7.

**Conclusion:**

This study showed that PAMAM dendrimer G5/ siRNA could be a useful system delivery for future gene therapy approaches.

## Introduction

Cancer is one of the most important causes of death in the world, and breast cancer is the second cause of death (1). Ectopic and high expression of OCT4, SOX2, KLF4, and c-MYC could reprogram somatic differentiated cells to an embryonic-like or pluripotent state (2-5). Long non-coding RNAs (lncRNAs) have important roles in metabolism and functional regulatory in all cells, including normal and cancer cells (3, 6-10). The most important group of lncRNAs are long intergenic non-coding RNAs (lincRNAs), which play major roles in regulating various cell functions and development of different diseases, including stem cell state and cancer metastasis (11-13). LincRNA-RoRs are involved in diverse functions, including stem cell pluripotency, sponges of miRNA (14). Linc-RoR possesses a binding site for pluripotency transcription factors (TFs) Oct4, Sox2, and Nanog, which competing endogenous RNA (CeRNA) to regulate the expression of nuclear transcription factor (4). Ectopic and high expression of lincRNA-ROR are observed in various cancers, including breast, hepatocellular, endometrial (15-21).

Poly (amidoamine) (PAMAM) dendrimers are effective vectors for siRNA delivery (22-23). *WDR7* gene encodes a member of the WD-repeat protein family. WD repeats are areas with the repetitions of approximately 40 amino acids that have ends with trp-asp and gly-his amino acids (GH-WD) which may help to form a multi-protein complex. WDR7 associates with rabconnectin 3 A and interacts with DMXL2; It plays a role in V-ATPase function (24). *WDR7* is located in 18q21.31 near the lincRNA-RoR (25).

For better understanding of regulatory effects of lincRNA-RoR in molecular biology and its role in cancer; they may lead to a novel cancer therapeutically approach. Which one of the combination of a long non coding RNA, siRNA and other gene therapies or chemical drugs are more efficient treatments than chemical therapies. Therefore, in this study focused on the effect of lincRNA-RoR silencing on gene expression level of the WDR7 gene. 

## Materials and Methods

Polyamidoamine (PAMAM) dendrimers were supplied from Sigma Chemical Co (USA). Roswell Park Memorial Institute medium (RPMI) purchased from Biosera. Fetal bovine serum (FBS) was obtained from Biosera. Antibiotics (10 000 units penicillin, 10 mg streptomycin) were supplied by (Sigma, St Louis, MO). The TriPure reagent was acquired from (SIGMA, USA). RNase-free DNase I was acquired from (Takara, Japan). 

***Gel shift assays***

Gel Shift Assays of siRNA/ PAMAM dendrimer complexes were performed by 1.2 % agarose gel. Two microliters of the siRNAs aqueous solution (100 ng/μL) with the desired amount of G5 PAMAM dendrimer solution were mixed gently and balanced at 37 ^°^C for 30 min to obtain various N/P ratios.

***Cell culture***

The human MDA-MB-231 breast cancer cell line was cultured in RPMI 1640 medium (Thermo Fisher Scientific) supplemented with 10% FBS and penicillin–streptomycin solution at 37 ^°^C in a humidified atmosphere with 5% CO_2_. 

***Transfection of siRNAs***

Two different linc-RoR siRNA (50 to 30); siRNA linc-ROR-1: GGAGAGGAAGCCTGAGAGT, and siRNA linc-ROR-2: GGTTAAAGACAC AGGGGAA as well as a non-targeting (NT) control siRNA (siGENOME Non-Targeting siRNA) transfected into MDA-MB-231 cell line with PAMAM G5 dendrimer without FBS serum in the cell culture (23).

***Cytotoxicity assays***

The effect of (siRNA-PAMAM dendrimers) dendriplex on the viability of MDA-MB-231 cells, was determined by using the MTT (3-(4,5-dimethylthiazol-2-yl)-2,5-diphenyltetrazolium bromide) assay. MDA-MB-231 cells were seeded at 1×10^4^ cells per well in a 96-well plate in a final volume of 200 µl/well at ~24 hr before the assay. Cells were exposed to different complexes of siRNA-PAMAM dendrimers at various concentrations without FBS serum. At the end of the incubation time (24 hr), 20 µl/well MTT reagent added to each sample and the plates incubated for 5 hr at 37 ^°^C in standard culture conditions. Finally, the absorbances measured at 490 nm using ELISA reader (Awareness Technology ChroMate® Microplate Reader). The estimated percentage of cell viability compared with the value of the untreated control cells.

***Real-time PCR analysis***

The total RNA was extracted from cells using Tripur (Roch) according to the manufacturer’s instruction. The RNA concentration was measured using Pico Drop A260/A280. To detect and compare gene expression, RNA was treated with RNase-free DNase I and cDNA was synthesized using Synthesis Kit (Takara). qRT-PCR was accomplished by Cyber Green Real-Time PCR Takara kit in Applied Biosystem 7300 Fast System (ABI; Foster City, CA, USA). GAPDH was used as an internal control. The ΔΔCt method (2-ΔCt) were used to characterize the level of gene expressions. The following PCR primers were applied: lincRNA-ROR primers, forward: 5’-ACAAGGAGGAAAGGGCTGAC-3’, Revers: 5’-TTCTGGAAGCTAAGTGCACATG-3’, and WDR7 primers, forward: 5’-AAAGTGGAGAGATGTGCCTCT-3’, reverse: 5’ AAA GCCTTCC TTCTCGCTGAT-3’.

***Statistical analysis***

Statistical analysis performed with GraphPad Prism 6 software. Results declared by mean±SD; One-way ANOVA and Student’s t-test used for statistical significance among study groups and *P-values*<0.05 were considered statistically significant.

## Results

***Gel retardation of nano-siRNA complexes***

The assembly of dendrimers-siRNA complex was confirmed by gel retardation. Anionic siRNA oligonucleotides was encapsulated after being mixed with polycationic nanocarriers; thus, inhibition of electrophoretic mobility of siRNA could be observed. In PAMAM-siRNA, band shift was blocked at an N/P ratio higher than 8. This result reveals that the best ratio of this complex is 10.

***Cell survival***

**Figure 1:**
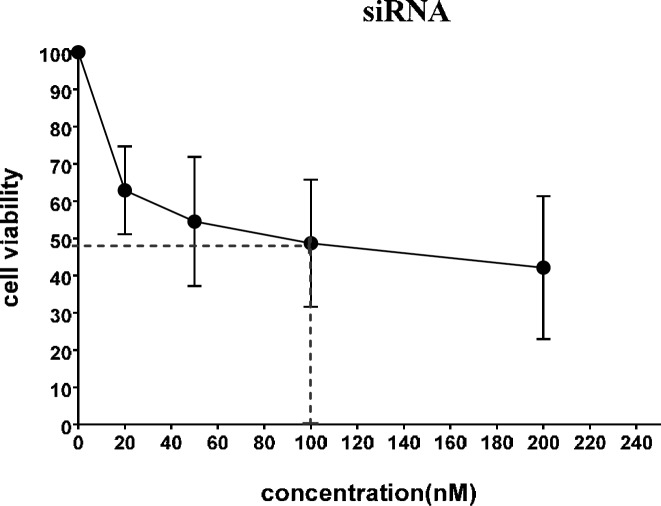
Cell toxicity assay of siRNA-PAMAM concentrations on MDA-MB-231 cancer cells. Three independent experiments have been performed with three replicates

MTT assay was made on MDA-MB-231 cells which were incubated with various concentrations of the same composition. Relative cell viabilities in 24 hr were calculated (Figure 1) and revealed the half-maximal inhibitory concentrations (IC_50_ value) of lincRNA-ROR siRNA was about 100 nanomolar. (*P*<0.05).

Based on the cell toxicity result siRNA concentration of 100 nM was selected for further *in vitro* transfection experiments.

***Gene expression analysis***

**Figure 2:**
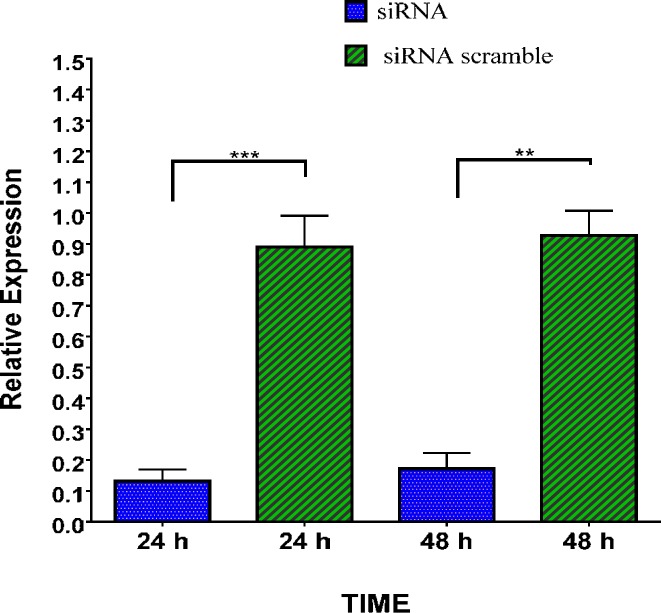
Changes in lincRNA-RoR gene expression at 24 and 48 hr after treatment with lincRNA-RoR siRNA

Small interfering RNA (siRNA) for linc-RoR was schemed; afterward the effect on linc-RoR siRNA was evaluated by qPCR. LincRNA-RoR gene expression level in MDA-MB-231 cells decreased after RoR siRNA transfection by-PAMAM Dendrimers. According to outcomes, the lincRNA-RoR gene expression significantly down-regulated compared to the cells treated with scrambled siRNA, as control (*P*-value= 0.02) (24 and 48 hr). The result represented an efficient siRNA transfection facilitated by PAMAM dendrimers and consequently, a successful gene silencing (Figure 2).

**Figure 3:**
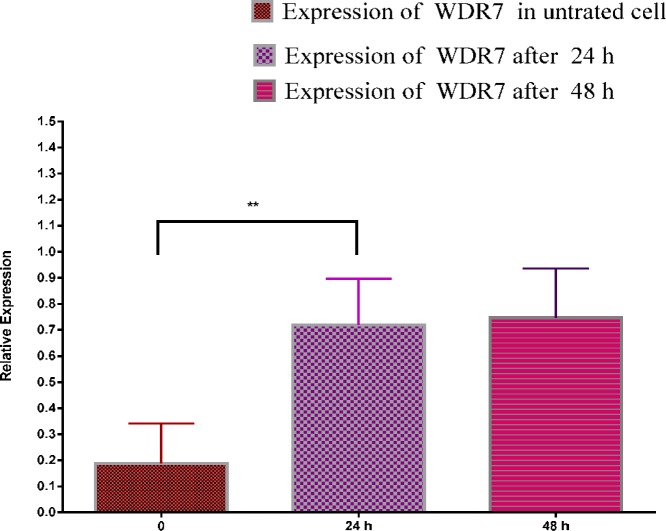
Relative expression of WDR7. Gene expression levels of WDR7 relative to the lincRNA-RoR. The comparison has been made before suppression 24 and 48 hr after the suppression of lincRNA-RoR expression

***The overexpression of WDR7 after suppressing of lincRNA-RoR***

WDR7 gene expression was compared with lincRNA-RoR expression before and after the suppression of lincRNA-RoR. WDR7 and lincRNA-RoR gene expression level comparison in MDA-MB231 cells were not considered according to the formula 2 ^ - (Δct) and have been calculated based on a comparison of normalized gene expression levels (26). WDR7 normalized expression relative to normal expression of lincRNA-RoR is low in the untreated cells (*P*-value = 0.0033), but after the suppression of lincRNA-RoR (24 and 48 hr), WDR7 expression was significantly increased (*P*-value = 0.002) (Figure 3).

## Discussion

According to the literature review, this is first report of lincRNA- RoR gene silencing in the MDA-MB-231 breast cancer cell line. lincRNA-RoR is a non-coding intergenic RNA, that was first obtained from stem cells. The non-coding RNA is involved in cancer through different mechanisms, including P53 pathways and reprogramming(17). It should be noted that according to Wang *et al.* lincRNA-RoR may regulate stem cell factors, including expression of Nanog, Oct4, and SOX2 (4). Though cancer stem cell theory indicates the origin of a cancer stem cell reins the growth of cancer stem cells and potentially can prevent cancer (27-28). Note that the purpose of molecular interposition is to reduce the expression of these molecules and reverts to the lincRNA-RoR, which has a role in maintaining pluripotency property (4). Nagano and Fraser discussed about the strong response of lincRNA-RoR to OCT4 knockdown, and their role in reprogramming in fibroblasts. Knockdown of lincRNA-RoR lead a significant decrease in induced pluripotent stem cells (iPSC) colony formation, which shows lincRNA-RoR plays a role in iPSC derivation (29). 

According to former researches, lincRNA-RoR overexpression in breast cancer is the cause of cancer progression, metastasis and tumor growth (17); But with our experience, this is first report of lincRNA- RoR gene silencing in MDA-MB-231 breast cancer cell line. 

Gene therapy in recent decades got more attention as a promising method to treat cancer and genetic disorders. RNAi is used for gene therapy and gene silencing, especially in research laboratories due to its high efficiency. Significant progress has been made in the field of siRNA nanoformulation, but still, there are no drugs on the market based on siRNA. An important issue is identifying safe and effective vectors to the successful delivery of siRNA and RNAi processes. Non-viral vectors also increasingly are proposed as alternatives to viral vectors, due to safety issues and the negative impact of viral methods for the delivery of siRNA (30). Non-viral vectors are considered dendrimer-based siRNA vectors (31). In this study, amidoamine dendrimer (PAMAM) G5 was used as a delivery vehicle for siRNA molecules into the MDA-MB-231 cell line. For the first time, lincRNA- RoR gene silencing in breast cancer cell lines showed increased delivery efficiency of the siRNA with PAMAM dendrimers to breast cancer cell line MDA-MB-231. Real-time PCR indicates decreased expression of lncRNA-RoR in MDA-MB 231 cell line. The exact functions of lncRNA-RoR and WDR7 genes and their relative expressions in various cancers are not clear. However, it was demonstrated that the WD protein family has roles in various processes of the cell, including cell cycle progression, signal transduction, apoptosis, regulation of gene, and human diseases. Although understanding the molecular processes and exact functions of WD proteins is a big obstacle (32). It can be inferred that abnormal expression of WDR7 is involved in cancer. As is shown non-coding RNA of the human genome is involved in regulating the expression of other genes. On the other hand, the location of WDR7 gene on chromosome 18 is near the lincRNA-RoR gene. Probably the positional effect of this gene is essential, and it can be explained that lncRNA adjacent to a gene with influencing on its expression has a regulatory effect (33). As mentioned earlier, the expression levels of linc-ROR and WDR7 genes are different. This study also indicates that the expressions of WDR7 and lincRNA-RoR in MDA-MB-231 breast cancer cell line are negatively related. In cell cycle and apoptosis, the expression of the lincRNA-RoR gene increases in breast cancer probably by reducing the expression of WDR7. 

## Conclusion

This study illustrated that PAMAM dendrimer G5/ siRNA can be a useful system delivery for future gene therapy approaches. The application of generation five of PAMAM dendrimer was efficient to transfect lincRNA-RoR siRNA. Furthermore, this study showed that the expression of the wdr7 gene is influenced by the lincRNA-RoR, probably as a regulatory effect of long non coding RNA mechanisms. More future study about lincRNA-RoR molecular biology and its role in cancer may lead to a novel cancer therapeutically approach in which combination of lincRNA-RoR siRNA and other gene therapies or chemical drugs are more efficient treatments than conventional and prevalent chemical therapies. 

## Conflicts of Interest

The authors declare that there are no conflicts of interest. 
